# Endothelial Dysfunction in Experimental Models of Arterial Hypertension: Cause or Consequence?

**DOI:** 10.1155/2014/598271

**Published:** 2014-03-13

**Authors:** Iveta Bernatova

**Affiliations:** Centre of Excellence for Examination of Regulatory Role of Nitric Oxide in Civilization Diseases, Institute of Normal and Pathological Physiology, Slovak Academy of Sciences, Sienkiewiczova 1, 813 71 Bratislava, Slovakia

## Abstract

Hypertension is a risk factor for other cardiovascular diseases and endothelial dysfunction was found in humans as well as in various commonly employed animal experimental models of arterial hypertension. Data from the literature indicate that, in general, endothelial dysfunction would not be the cause of experimental hypertension and may rather be secondary, that is, resulting from high blood pressure (BP). The initial mechanism of endothelial dysfunction itself may be associated with a lack of endothelium-derived relaxing factors (mainly nitric oxide) and/or accentuation of various endothelium-derived constricting factors. The involvement and role of endothelium-derived factors in the development of endothelial dysfunction in individual experimental models of hypertension may vary, depending on the triggering stimulus, strain, age, and vascular bed investigated. This brief review was focused on the participation of endothelial dysfunction, individual endothelium-derived factors, and their mechanisms of action in the development of high BP in the most frequently used rodent experimental models of arterial hypertension, including nitric oxide deficient models, spontaneous (pre)hypertension, stress-induced hypertension, and selected pharmacological and diet-induced models.

## 1. Introduction

Cardiovascular diseases account for about one-third of premature deaths in men and one-quarter of premature deaths in women, and arterial hypertension is one of the most significant risk factors for cardiovascular diseases. Despite current knowledge and extensive clinical and experimental research, the cause of hypertension remains unknown in about 95% of all cases. There are several factors that—alone or in combination—can increase the risk of developing primary hypertension in humans. In general, these include genetic and environmental factors.

Genetic association studies have identified polymorphisms in several candidate genes (e.g., angiotensinogen, angiotensin-converting enzyme, alpha-adducin, beta-adrenergic receptors, endothelial nitric oxide synthase (NOS), cytochrome P_450_ 2C19, and nicotinamide adenine dinucleotide phosphate oxidase (NADPH oxidase)) and several genomic sites that may include other genes contributing to primary hypertension [1–11]. However, none of these genetic abnormalities seems to be responsible for a significant portion of hypertension in the general population. Yet the influence of genetic factors may be accentuated and disease can be triggered by interaction of several gene polymorphisms or with environmental inputs such as sedentary life style, smoking, dietary factors (high salt, sugar, fat/cholesterol and alcohol intake, and low potassium and calcium intake) and chronic stress.

It is well known that pathophysiological characteristics of essential hypertension involve, besides other factors, increased total peripheral resistance. Thus, several experimental models of hypertension have been developed in rodents to study the mechanisms of blood pressure (BP) regulation in order to better understand the cause and consequences of human arterial hypertension [12]. These experimental models allow not only to modify all potential factors—diet, surrounding environment, and genetic information (by using specific gene knock-out or transgenic models)—but also to study the influence of interaction of specific risk factors in the etiology of hypertension. Moreover, these models are developed to allow investigation of endothelial function of various arteries, either* in vivo* or* in vitro*, including determination of mechanisms involved in loss of normal vascular function.

Endothelium, the inner layer of the blood vessels, was originally considered to be a passive barrier between blood and the vascular wall. This opinion was broken after the discovery of prostaglandin X by Bunting and coworkers in 1976 [13]. Their study showed that the arterial wall can synthetize and release a vasoactive substance that is able to relax arterial strips and to inhibit platelet aggregation. Prostaglandin (PG) X was soon identified as PGI_2_ (prostacyclin) synthetized and released by the endothelial cells [14]. However, only the discovery of the obligatory role of the endothelium in relaxation of the arterial wall by the chemically unknown “endothelium-derived relaxing factor (EDRF)” by Furchgott and Zawadski in 1980 [15] and identification of EDRF as nitric oxide (NO) started the real age of “endothelial research.” Soon it became clear that prostacyclin and NO are not the only vasoactive substances released by the endothelium.

Today it is known that the endothelium produces various substances collectively termed endothelium-derived relaxing factors (EDRFs) and endothelium-derived constricting factors (EDCFs), based on their function in modulation of the arterial wall. In addition to these factors there are vasorelaxing endothelium-derived hyperpolarizing factors (EDHFs), whose chemical moiety is still under discussion. This brief, though not exhausting, review is focused on main mechanisms involved in the development of endothelial dysfunction in selected models of experimental hypertension.

## 2. Endothelium-Derived Factors

### 2.1. Endothelium-Derived Relaxing Factors

NO, PGI_2_, and hydrogen sulfide (H_2_S) were described as EDRFs and NO is the best characterized EDRF. In addition, there is a group of EDRFs that produce relaxation due to hyperpolarization and they all go under the term EDHFs.

**Figure 1:**
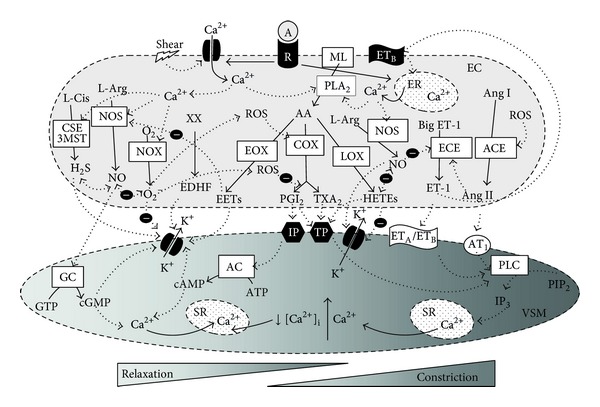
A brief scheme of interactions among the individual endothelium-derived factors and their mechanisms of action in the endothelial cells. Abbreviations are explained in the list of abbreviations. The activation of the appropriate receptor by its agonist as well as shear stress leads to alterations in the intracellular calcium concentration in the endothelial cells which affect the activity of all NOS, CSE, NOX, and PLA_2_ resulting in the release of NO, H_2_S, ROS, and AA-derived metabolites, respectively. ROS can further inhibit (marked by “−” sign) the production of PGI_2_ and to elevate TXA_2_ and Ang-II production. In addition, there are significant interactions among the NO, H_2_S, and superoxide as well as among NO, Ang-II, ET-1, and HETEs. Individual EDRFs then affect the vascular smooth muscle cells, via modulation of the appropriate receptors or channels, resulting in the respective vascular smooth muscle cell relaxation and constriction.

In mammals NO is produced by one of four nitric oxide synthase (NOS) isoenzymes (nNOS/NOS I, that is, neuronal NOS; iNOS/NOS II, that is, inducible NOS; eNOS/NOS III, that is, endothelial NOS; or mtNOS/NOS IV, that is, mitochondrial NOS). They are involved in the modulation of various pathophysiological or diseased states. Endothelial NOS is the main isoform expressed in the endothelium. It is localized in cellular plasma membranes in the caveolae and in the membrane of the Golgi body [16]. Briefly, the regulation of NO production in the endothelium depends on bioavailability of several cofactors, phosphorylation of eNOS in specific sites, co- and posttranslational lipid modifications, and it is a subject of negative feedback regulation by NO itself [17–20]. NO can diffuse from endothelial cells to vascular smooth muscle cells and guanylate cyclase (GC) has been identified as an intracellular receptor for NO (Figure 1). Its activation leads to the release of the second messenger cyclic guanosine monophosphate (cGMP) and activation of protein kinase (PK) G-dependent mechanisms resulting in reduction of intracellular calcium concentration ([Ca^2+^]_i_), followed by vasorelaxation. In addition, there are many other functions of NO participating in the regulation of gene transcription, mRNA translation, and protein modification of various enzymes involved in mitochondrial respiration, mitogenesis, and growth [19, 21, 22].

As mentioned above, PGI_2_ was the first endothelium-derived relaxing factor discovered. PGI_2_ is the main product of arachidonic acid (AA), cyclooxygenase- (COX-) mediated metabolism in vascular tissues. In normal conditions, PGI_2_ produced by prostacyclin synthase acts on the prostacyclin receptor (IP). Activation of the IP receptor leads to G protein-coupled increase in the second messenger cyclic adenosine monophosphate (cAMP) and PK A activation, resulting in decreased [Ca^2+^]_i_ and vasorelaxation. In the vasculature, PGI_2_ inhibits cell adhesion, thrombosis, inflammation, apoptosis, and proliferation. It participates in vasorelaxation and BP regulation; however, its role in these functions is rather minor as compared to NO [23, 24].* In vitro* studies have suggested that there is a cross-talk between NO and PGI_2_ production as NO was shown to activate the enzymes involved in PGI_2_ synthesis and vice versa [25, 26].

Hydrogen sulfide can be produced in the endothelial cells by cystathionine *γ*-lyase (CSE) and 3-mercaptopyruvate sulfur esterase (3MST) [27, 28]. Genetic deletion of CSE in mice markedly reduces H_2_S levels in the aorta and other tissues. Mutant mice lacking CSE display diminished endothelium-dependent vasorelaxation after muscarinic cholinergic stimulation of vascular endothelial cells and pronounced hypertension. CSE is physiologically activated by calcium-calmodulin, which is a mechanism for H_2_S formation in response to vascular activation [27].

### 2.2. Endothelium-Derived Hyperpolarizing Factors

Although many substances produced by the endothelium act as EDHFs, there is continuous discussion on the chemical moiety and molecular signaling of individual hyperpolarization-producing factors. Substances like NO, hydrogen peroxide (H_2_O_2_), carbon monoxide, adenosine [29], and K^+^ itself [30] possess the ability to induce hyperpolarization. Furthermore, recently published studies showed that H_2_S yielded significant hyperpolarization of vascular smooth muscle cells [31, 32].

In addition to these factors, several authors included epoxyeicosatrienoic acids (EETs) in EDHFs. EETs are synthesized in endothelial cells in the AA pathway, in which AA is converted by cytochrome P_450_ (CYP) epoxygenases to 5,6-EET, 8,9-EET, 11,12-EET, and 14,15-EET. The targets of EETs are large conductance calcium-activated K^+^ channels (BK_Ca_) in vascular smooth muscle cells, as well as small (SK_Ca_) and intermediate (IK_Ca_) conductance K_Ca_ channels of endothelial cells, whose activation leads to hyperpolarization [33]. Besides regulation of vascular tone, EETs participate in vascular signaling processes involved in inflammation and angiogenesis.

The importance of individual EDHFs may vary among various vascular beds and animal species. Regarding the issue of artery size, EDHF-mediated relaxation is a dominant component of acetylcholine- (ACh) induced relaxation in the small arteries, while its contribution to relaxation of the aorta is minor [23]. Differences in the mechanism of ACh-induced relaxation were observed also in mice with disruptions in the NOS genes, that is, eNOS^−/−^, n/eNOS^−/−^, and n/i/eNOS^−/−^ mice [34]. Studies of Takaki et al. [34] revealed that EDHF-mediated relaxation and hyperpolarization in response to ACh in the small mesenteric arteries of mice were progressively reduced as the number of disrupted NOS genes increased, whereas vascular smooth muscle function was preserved, suggesting that loss of eNOS expression alone was compensated by other NOS genes. However, EDHF/H_2_O_2_-mediated responses were completely absent in n/i/eNOS^−/−^ mice. Studies showed that NOS isoforms (nNOS, iNOS, and eNOS), especially eNOS, produce both NO and superoxide anions, and the latter is dismutated by superoxide dismutase (SOD) to EDHF/H_2_O_2_, which elicits hyperpolarization followed by vasodilation. In addition, the authors showed that NOS uncoupling was not involved in reactive oxygen species (ROS) production, as modulation of tetrahydrobiopterin (BH_4_) synthesis had no effect on EDHF-mediated relaxation, and the BH_4_/dihydrobiopterin ratio was comparable in the small mesenteric arteries and the aorta. Collectively, studies of Takaki et al. [34] provided a novel concept on the diverse roles of endothelial NOS system mainly contributing to the EDHF/H_2_O_2_ responses in microvessels while serving as NO-generating system in the large arteries.

The ratio of NO-dependent and NO-independent ACh-induced relaxation in various vascular beds is, in addition to artery size, affected by the cardiovascular genotype as well as by age. We found that NO-dependent and independent components of ACh-induced relaxation were approximately equal in the femoral artery of young (7 weeks' old) normotensive male Wistar-Kyoto (WKY) rats, while NO-dependent relaxation was dominant in age-matched spontaneously hypertensive rats (SHR). Furthermore, aging reduced NO production and NO-dependent relaxation in both normotensive and hypertensive rats [35–37].

### 2.3. Endothelium-Derived Constricting Factors

The endothelium can produce many constrictor factors including endothelin-1 (ET-1) and angiotensin II (Ang II), which appear to be the most powerful constrictors in the vasculature. Data in the literature suggest that ET-1 acts as mediator of Ang II-produced vasoconstriction [38–40].

However, experimental studies showed that significant effects of EDCFs in hypertensive rats were associated with COX products such as thromboxane A_2_ (TXA_2_), prostaglandins H_2_, E_2,_ and F_2*α*_, isoprostanes, and monohydroxyeicosatetraenoic acids (HETEs), whose effect is associated mainly with thromboxane/prostaglandin endoperoxide receptor (TP) [41, 42]. EDCF-induced responses can be eliminated, at least partially, by COX-2 inhibitors and TP receptor blockers [43–45]. Interestingly, in conditions of lack or malfunction of the IP receptor, PGI_2_ can produce contraction via the TP receptor both in young [46] and aged [47] rats.

Furthermore, ROS such as H_2_O_2_, hydroxyl anion, and mainly superoxide were shown to constrict the arteries by modulation of the action of other endothelium-derived factors. ROS can be produced by mitochondria, uncoupled NOS, and various oxidases—xanthine oxidase, COX, lipoxygenases (LOX), and CYP monooxygenases. Yet NADPH oxidase was shown to serve as a primary source of so called “kindling radicals” [48]. The accentuated release of ROS is considered the main factor involved in vascular aging [37]. On the other hand, optimal ROS production is required for normal cell signaling as ROS serve as second messengers involved in activation of nuclear factor kappa B (NF-*κ*B) and further in regulation of mitogen-activated protein kinase (MAPK) pathways including extracellular signal-regulated kinases 1 and 2 ERK1/2, p38 mitogen-activated protein kinase (p38MAPK), c-Jun N-terminal kinase (JNK), and extracellular-signal-regulated kinase 5 (ERK5), with their respective importance in cell growth, inflammation, apoptosis, and cell differentiation [49–52].

In the vasculature, ROS possess pleiotropic effects which might be variable depending on the oxidative status of the tissue. For example, H_2_O_2_-induced oxidative stress increased vascular TP sensitivity and predisposed segments of the small arteries to prostanoid-induced constriction. Conversely, H_2_O_2_-induced vasodilation was observed in the same segments in the presence of antioxidants targeting radicals downstream of H_2_O_2_ [53].

Thus, correct function of the endogenous antioxidant defense systems, in association with the exogenous antioxidants, is necessary for the maintenance of balance between EDRFs and EDCFs and for normal vascular function.

## 3. Endothelial Dysfunction and Its Classification

Besides the balance between ROS and NO, there is complex cross-talk among the individual endothelium-derived factors with the aim to maintain appropriate endothelial function (Figure 1). Dysregulation of this cross-talk can result in alteration of normal physiological processes carried out by the endothelium, including reduction of its anticoagulant and antithrombotic properties, acceleration of vascular growth and remodeling, and impairment of endothelium-dependent vasorelaxation, that is, in endothelial dysfunction (ED). Yet neither the factors and mechanisms that modulate the balance between relaxing, anticoagulant, antithrombotic, and anti-mitotic factors on the one side and constricting, proaggregatory, and promitogenic factors on the other, nor the processes in which endothelium loses its protective functions have been fully elucidated so far. Genetic predisposition and aging [35, 54], several environmental factors such as sedentary life style, smoking, improper diet, and stress might participate in the transformation of the endothelium from a protective to a “health-threatening” organ.

In the last two decades, enormous research on ED has been conducted; however, the issue whether ED is a cause (i.e., primary) or a consequence (i.e., secondary) of high BP remains still open. In fact, the increasing, but still not exhausting, knowledge on the etiology and pathophysiology of ED suggests the need of proper classification of this disorder that could facilitate the integration of current state of the art in this field. Such classification has been proposed by Evora [55] and it is briefly described in Table 1. It includes etiological, functional, and evolutionary or prognostic aspects of ED. It should however be noted that the proposed classification has not yet been widely discussed, opposed, or accepted among scientists. As mentioned later in this review, a correct etiological classification of ED might be very difficult in some experimental models of hypertension. Moreover, ED may occur in other diseased states independently of hypertension [56].

## 4. Endothelial Dysfunction in Experimental Hypertension

### 4.1. Nitric Oxide-Deficient Models of Hypertension

One of the first diseases associated with reduced bioavailability of EDRF and altered vascular function was arterial hypertension. The importance of endogenous eNOS-derived NO production in regulation of vascular function underwent increasing investigation with implementation of NOS inhibitors. Several studies reported elevation of BP after intravenous administration of NOS inhibitors in rabbits [57], guinea pigs [58], dogs [59], monkeys [60], and rats [61, 62]. The increase in BP was confirmed also during long-term oral treatment with NOS inhibitors [63–67]. The experimental model of “N^G^-nitro-L-arginine methyl ester (L-NAME)-induced” or “NO-deficient” hypertension [68] was established to investigate not only the role of NO in vascular function and BP regulation but also in maintenance of homeostasis in the whole cardiovascular system.

Briefly, the complex mechanisms responsible for BP increase in this model of hypertension involve attenuated vascular relaxation and increased contraction in different parts of the vascular tree [69–72]. In addition, chronic NOS inhibition increases endothelium-dependent contractions of the rat aorta by inducing COX-2 expression and augmenting the production of EDCFs [73, 74] as well as by accentuation of ET-1 effect. The complex mechanism of BP increase in NO-deficient hypertension involves also accentuation of the sympathetic system tone, the renin-angiotensin system, and oxidative stress [75–78]. Furthermore, NO can modulate vascular remodeling independently of BP, which contributes to the maintenance of high BP [21, 79].

In the NO-deficient model of experimental hypertension, due to chronic nonspecific inhibition of NO production, the development of ED is associated with a gradual elevation of BP. Thus, regarding the abovementioned classification, ED in this model of hypertension might be classified as primary. Indeed, functional and morphological alterations observed in the model of L-NAME-induced hypertension [79] were similar to those observed in mice, in which three NOS isoforms were disrupted, that is, n/i/eNOS^−/−^ [80]. Moreover, studies that used triple NOS disrupted n/i/eNOS^−/−^ mice showed that the magnitude of hypertension in the triply NOS^−/−^ mice was similar to that in mice with the eNOS gene disrupted singly (eNOS^−/−^) or doubly (n/eNOS^−/−^ or i/eNOS^−/−^) [81]. Besides hypertension, ED due to lack of NO has been shown to be involved also in other pathological states. For example, mice with single disruption of the eNOS gene (eNOS^−/−^) were insulin resistant, displayed increased triglycerides and free fatty acid levels, defective mitochondrial *β*-oxidation, and renal dysfunction [82–85].

The abovementioned studies suggest that hypertension is a common characteristic of the lack of eNOS-produced NO. Moreover, studies pointed out the association between eNOS-derived NO, primary ED, hypertension, and metabolic disorders involved in the cluster of metabolic syndrome as well as the development of atherosclerosis. Indeed, elevated BP and altered vascular function were observed in various genetic and diet-induced models of metabolic syndrome [86].

### 4.2. Models of Spontaneous Genetic Hypertension

Contrary to the NO-deficient model of hypertension, studies experimenting with the genetic model of spontaneously hypertensive rats produced inconsistent results regarding the role of NO and ED in the development of hypertension. The findings of ED in SHR depend on many factors, such as age, artery type, and methods used for determination of vascular function. A similar inconsistency was observed in NO production in SHR, in which reduced [87, 88], unchanged [89], and elevated [90–94] vascular NO synthesis and/or NOS expression were observed. Our previous reviewing of the literature showed that the enormous variability in the results concerning ED in SHR might result, on the one hand, from various methodologies used for determination of vascular function. On the other hand, regardless of methodology, aging seems to be an important factor in studying ED. ED was observed mainly in adult and aged (older than 25 weeks) and not in young (less than 6 weeks' old) SHR [95]. In our studies we observed elevated NOS activity in the aorta also in borderline hypertensive rats (BHR), which were the first filial generation of offspring of one normotensive and one hypertensive parent. In these rats, borderline hypertension (with systolic BP about 140 mmHg) was recorded in adulthood [92]. We also determined positive correlation between BP and NO-dependent component of ACh-induced relaxation in the femoral artery in adult rats [96]. We revealed that ED in the femoral artery in adult BHR and SHR males resulted rather from decreased NO-independent relaxation than from a lack of NO, a finding suggested also by other authors [47, 97]. In addition, we observed a decreased NO-independent component of ACh-induced relaxation already in young (7 weeks' old) male and female SHR [36, 98]. Interestingly, in young BHR and SHR females, ED was underlined also by a decrease of the NO-dependent component of relaxation and elevated superoxide production, which was not observed in males, suggesting sex-related differences in the mechanism of ED development.

To elucidate the causal relation between ED and high BP, we used young BHR rats whose BP is significantly elevated versus WKY as early as at the age of 7 weeks. However, despite higher BP and vascular ROS production, we did not observe ED in these rats as reduced NO-dependent relaxation was fully compensated be elevated NO-independent relaxation [98]. Thus, our findings in young BHR rats are not supportive of the idea that ED precedes hypertension in this particular model of hypertension. According to the suggested ED classification, ED in BHR (and supposedly also in SHR) seems to be rather secondary and other nonendothelial mechanisms are responsible for the induction of BP increase.

However, elevated BP itself can trigger damage of endothelial function and vascular remodeling [99]. Chronic presence of high BP* per se *was found to elicit increased arterial superoxide production by activating directly a PKC-dependent NADPH oxidase pathway, but also, in part, via activation of the local renin-angiotensin system [100].

Regarding the role of ROS in the development of hypertension, in addition to the reduction of bioavailable NO, ROS may also reduce hyperpolarization and PGI_2_ synthesis [35]. Elevated ROS production was observed in the aorta of young BHR and SHR [98] as well as in adult SHR [45, 101].

Recently, the role of excessive NO production by iNOS has been suggested in the development of ED. Elevated expression of iNOS was observed already in young SHR rats but not in WKY and inhibition of iNOS prevented BP increase [87, 102]. Yet whether iNOS activation participates in ED development has to be further investigated as normal iNOS expression was detected in the small mesenteric arteries from SHR [45]. However, in association with high NO production observed in SHR, elevated ROS may result in formation of peroxynitrite, thereby promoting nitrosative stress and ED. The interaction between NO and superoxide occurs at an extremely rapid rate of 6.7 × 10^9^ mol/L^−1^·s^−1^ and is about 3-times faster than the reaction rate for superoxide with SOD [103].

ROS have been implicated also in the mechanism of AA-derived EDCFs-mediated development of ED in genetic hypertension due to activation of COX [104]. ACh-induced constrictions were observed in adult SHR and BHR as well as in aged WKY [105]. As ACh led to release of PGI_2_, PGH_2_, as well as PGE_2_ and PGF_2*α*_ acting via TP receptors in the aorta [46, 105], all these substances may be involved in EDCF-induced ED in SHR. The contribution of EDCFs to ED was determined also in other arteries (mesenteric, renal, basilar, carotid, and others), yet the nature of EDCFs might be different from that observed in the aorta [105].

Regarding the mechanism of triggering elevated EDCFs release, accentuated accumulation of calcium in the endothelial cells was found to be an initial prerequisite. As intracellular calcium is required also for eNOS [106] and NADPH oxidase activation [107], it seems plausible that defective calcium handling or signaling in the endothelium [108], resulting in abnormal calcium accumulation, might account for the acceleration of EDCFs production resulting in ED. Then even mild ED, in association with increased calcium influx and sensitivity observed in the vascular smooth muscle cells of SHR [109], can markedly facilitate contractility and initiate BP increase.

### 4.3. Pharmacological and Diet-Induced Models of Hypertension

In addition to the abovementioned models of hypertension, there are various pharmacological and diet-induced models. These models allow investigating the involvement of specific factors (salt, corticoids, lead, sugar, etc.) and pathways which may affect endothelial function and thus trigger the development of hypertension. Although the exact disorders in signal transduction resulting in hypertension in the individual models of hypertension may not be known, involvement of oxidative stress has been suggested in Ang II-induced hypertension [110], ET-1-induced hypertension [111], Dahl salt-induced hypertension [112], deoxycorticosterone acetate- (DOCA-) salt induced hypertension [113, 114], fructose-induced [115, 116], and lead-induced hypertension [117].

In some models reduced NO bioavailability was determined as a consequence of elevated ROS [112, 117], yet in other models of hypertension the participation of NO deficiency in ED seems to be of rather minor importance.

Numerous mechanisms implicated in ED development were observed in the model of Dahl salt-sensitive rats. Regarding EDCFs (likely PGH_2_ and TXA_2_), their contribution to ED was observed in carotid rings [118]. Lukaszewicz and Lombard [119] demonstrated restoration of normal vascular function in Dahl salt-sensitive rats with inhibition of the CYP4A/20-hydroxyeicosatetraenoic acid pathway, suggesting a direct role for this pathway in vascular dysfunction. Involvement of the endothelin receptor type A (ET_A_)-mediated effect of ET-1 in the development of hypertension was observed in adult but not in young Dahl rats [120, 121]. Furthermore, high salt diet significantly reduced expression and activity of endothelial dimethylarginine dimethylaminohydrolase (DDAH-2), involved in asymmetric dimethylarginine (ADMA, endogenous NOS inhibitor) degradation and reduced eNOS expression, independently of BP [122]. Involvement of iNOS in modulation of endothelial function was not observed in salt-induced hypertension as W1400 (specific iNOS inhibitor) failed to modify aortic function [123]. Interestingly, in the same study NO produced by renal medullary iNOS was found to prevent excessive increases in arterial BP.

Endothelial dysfunction associated with increased aortic superoxide content, elevated NADPH oxidase activity, and decreased phosphorylated eNOS levels in aortic rings was observed also in DOCA-salt treated rats [114]. Similarly, elevated oxidative stress, yet due to activation of xanthine oxidase, was found in both the aorta and mesenteric arteries of DOCA-salt hypertensive rats in association with elevated expression of the ET_A_ receptor [113] and ET-1 gene [124]. Participation of elevated COX-2-derived factors that enhanced rat aortic contractility was determined in DOCA-salt treated rats by Adeagbo et al. [125]. Moreover, involvement of COX-2 mediated free radicals that impaired EDHF-mediated relaxation in the mesenteric arteries of DOCA-salt induced hypertension was also observed, while NO-dependent relaxation remained unaltered [126].

In the model of fructose-induced hypertension, the development of hypertension was associated with insulin resistance, hyperinsulinemia, and NO-dependent ED with preserved endothelium-independent vasomotion [127]. On the other hand, Lee et al. [128] did not observe reduced NO_*x*_ levels or protein expression of constitutive NOS and iNOS in the aorta of fructose-treated rats. However, their results indicate that an increased expression of vascular ET-1 may be causally related to the development of hypertension due to high fructose intake. This finding is in agreement with the observation of similar relaxing responses to ACh and sodium nitroprusside in the mesenteric arteries of fructose-treated rats in which accentuated Ang II-induced constriction was observed [129]. Finally, in contrast to salt-induced hypertension, in fructose-induced hypertension elevated ADMA levels seem to be secondary to the early development of NADPH oxidase-independent oxidative stress and elevated iNOS expression [116].

On balance, the findings in the abovementioned models of hypertension suggest that vascular NO deficiency would not be primarily involved in the hypertension development in Dahl salt-treated, DOCA-salt-treated, and fructose-treated rats while ET-1 seems to contribute significantly to ED in these models. Again, specific mechanisms can be involved in initial stages of ED in various arteries investigated.

### 4.4. Stress-Induced Hypertension

Stress is another important factor that has been suggested in the development of hypertension. Although the problem of stress-related hypertension has been addressed in several studies, there are still conflicting data regarding causal relation between stress and hypertension in humans [130, 131]. In animal studies, some models of psychosocial stress were able to induce hypertension in normotensive rats [132, 133], while other models did not produce changes in BP [134–136]. In addition, animal studies revealed the importance of genetic factors in the etiology of stress-induced hypertension [136–138], thus genetically consistent rats, either normotensive or SHR, may not be always suitable for investigation of cardiovascular effects of stress. The main mechanisms involved in BP elevation in stress are associated with sympathoadrenal activation and less information is available on the role of ED in stress-induced models of hypertension.

To investigate the influence of chronic stress on vascular function, we employed the model of chronic social stress produced by crowding resulting from reduced living space. This model seems to be more relevant to the human situation [139, 140] than other stress models.

In our studies chronic crowding stress failed to increase BP in normotensive rats, either by the use of 2-week crowding in young females or of 8- or 12-week crowding in adult male WKY. Interestingly, at all time points investigated, we always found elevated vascular NO production in the aorta of stress-exposed WKY [98, 141–143]. Despite the consistency in these findings, the effect on vascular function was variable. In adult males exposed to 8-week stress, endothelium-dependent relaxation was elevated versus control in both femoral and mesenteric arteries, which was associated with improvement of NO-dependent relaxation [143, 144]. Notably, maximal ACh-induced relaxation was reduced after 12 weeks, while NO-dependent relaxation was still elevated, suggesting the development of NO-independent ED in crowded WKY. Furthermore, 2-week stress reduced NO-dependent relaxation in the femoral artery in young WKY females, but this was fully compensated by increase of the NO-independent component [98].

In contrast to normotensive rats, the same stress model led to elevation of BP in SHR and SHR-mothered BHR but not in Wistar-mothered BHR after 8-week exposure. However, stress-reduced relaxation was seen only in SHR [92]. In young females, 2-week stress led to acceleration of time-related increase of BP in BHR yet relaxation in the femoral artery was not altered despite elevated NO production [98].

Fuchs et al. [145] observed alterations in the mechanisms mediating endothelium-dependent relaxation to ACh in small mesenteric arteries isolated from adult male WKY and BHR rats after 10 days of repeated air-jet stress. In their study, decrease of NOS activity had a significantly larger inhibitory effect on ACh-induced relaxation in arteries from stressed compared with control BHR. COX-derived products contributed to ACh-induced relaxation of the small mesenteric arteries from stressed WKY rats, but not control WKY rats or BHR [145]. The same stress model led also to impairment of coronary artery relaxation in BHR [146]. In addition, the effect of air-jet on ACh-induced relaxation of the coronary artery was altered with aging in BHR. In young adult (3 months' old) BHR males exposure to stress produced an NOS-dependent increase in relaxation, while a decrease in relaxation was observed in aged (18 months' old) BHR. The impaired response to ACh observed in aged BHR was associated with superoxide anion, vasoconstrictor prostaglandins, and a loss of the component of relaxation that was NO-independent and K^+^ channel mediated [147].

In 129/SV mice exposed to 28 days of stress (consisting of exposure to rat, restraint stress, and tail suspension) impaired carbachol-induced endothelium-dependent vasorelaxation, increased superoxide production, and reduced aortic eNOS levels were observed. These changes were reversed by the glucocorticoid (GCC) receptor antagonist mifepristone [148].

The involvement of GCC in disruption of NO production and/or expression was reported in studies using cultured endothelial cells. Radomski et al. showed that GCC inhibited the expression of an iNOS but not of constitutive NOS in cultured porcine vascular endothelial cells [149]. The inhibitory effect of GCC on eNOS expression and NO_*x*_ production was observed in cultured bovine coronary artery and aortic endothelial cells as well as in human umbilical vein endothelial cells [150, 151]. GCC was shown to downregulate NO production by limiting BH_4_ production also in cardiac microvascular endothelial cells [152]. Moreover, GCC response elements in the eNOS promoter region were demonstrated by Liu et al. [153]. In addition to modulation of NO, GCC was shown to downregulate COX-1 expression and PGI_2_ synthesis in the fetal pulmonary artery endothelial cells through activation of the glucocorticoid receptor (GR) and effects on COX-1 gene transcription [154].

Oxidative stress is another parameter that was shown to be increased by GCC. Decline in antioxidant defence by actions of corticosterone was evidenced by coordinate decreases in the activities of free-radical scavenging enzymes SOD, catalase, glutathione S-transferase, and glutathione reductase in the brain, liver, and heart of rats [155].

Surprisingly, in our studies in which elevated level of corticosterone resulted from chronic stress and not from pharmacological intervention, elevated corticosterone levels observed in adult WKY and young BHR and SHR failed to reduce NO-dependent relaxation compared to control rats [98, 143]. Our findings pointed out adapting mechanisms that play a role under chronic stress and may not be active in endothelial cell cultures and during acute stress exposure. We further considered the possible involvement of NOS isoforms different from eNOS.

NO has been previously suggested as a stress relieving molecule [156, 157] and activation of the vascular L-arginine/NO pathway may serve as an antistress system* in vivo*. Our results support the idea that elevated NO production in the vasculature can be considered one of the adaptation mechanisms protecting from sustained elevation of BP, at least in normotensive rats in allostasis. Long-term stress, however, may induce NO-independent ED that might be the initial step in the development of vascular remodelling followed by atherosclerosis and/or hypertension. Thus, in case of chronic stress-induced hypertension in genetically normotensive rats, NO-independent ED can precede hypertension and might be considered primary. Whether corticosterone is involved in the initiation of ED in stress-induced models of hypertension, or even in spontaneous hypertension, remains to be clarified.

## 5. Conclusion

The conclusion that can be drawn from reviewing the literature is that ED might be both a cause and consequence of high BP, depending on the model of hypertension used, strain, age, and vascular bed. The findings in the abovementioned models of hypertension suggest that ED, due to genetic or pharmacological disruption of eNOS-derived NO, observed before sustained elevation of BP, might be considered to be primary. Such ED is causally associated with reduced NO-dependent relaxation. On the other hand, ED observed in SHR and BHR seems to be EDCF associated and NO independent, and it can be considered to be secondary ED since numerous studies confirmed the presence of elevated BP before the development of ED. Furthermore, contribution of ET-1 to ED development was predominant in diet-induced models. Yet the most prevalent cause of ED seems to be oxidative stress that has been observed in all abovementioned experimental models of hypertension. As oxidative stress may result from a broad spectrum of genetic and environmental factors as well as from high PB* per se*, it is very difficult to distinguish between primary and secondary ED in experimental models of hypertension because the processes of endothelial function damage and elevation of BP are many times occurring simultaneously.

## Figures and Tables

**Table 1:** Classification of endothelial dysfunction proposed by Evora [55]. For more details and explanation see the original article.

Endothelial dysfunction classification	
(I) Etiological classification	
(A) *Primary or “genotypic”*: demonstrated for example in normotensive patients with familial antecedents of essential arterial hypertension.	
(B) *Secondary or “phenotypic”*: present for example in cardiovascular diseases including arterial hypertension.	
(II) Functional classification	
(A) *“Vasotonic”*: implying a risk of vasospasm and thrombosis.	
(B) *“Vasoplegic”*: associated with a pathological release of endothelium-derived relaxing factors.	
(III) Evolutionary or prognostic classification	
(A) *Reversible *	
(B) *Partially reversible *	
(C) *Irreversible *	

## Abbreviations

A: Agonist
AA: Arachidonic acid
AC: Adenylate Cyclase
ACE: Angiotensin-converting enzyme
ADMA: Asymmetric dimethylarginine
ACh: Acetylcholine
Ang I: Angiotensin I
Ang II: Angiotensin II
AT_1_: Angiotensin II receptor 1
ATP: Adenosine-5′-triphosphate
BH_4_: Tetrahydrobiopterin
BHR: Borderline hypertensive rats
BK_Ca_: Large conductance calcium-activated K^+^ channel
BP: Blood pressure
cAMP: Cyclic adenosine monophosphate
cGMP: Cyclic guanosine monophosphate
COX: Cyclooxygenase
CSE: Cystathionine *γ*-lyase
CYP: Cytochrome P_450_
DDAH-2: Endothelial dimethylarginine dimethylaminohydrolase
DOCA: Deoxycorticosterone acetate
EC: Endothelial cell
ECE: Endothelin-1-converting enzyme
ED: Endothelial dysfunction
EDCF(s): Endothelium-derived constricting factor(s)
EDHF(s): Endothelium-derived hyperpolarizing factor(s)
EDRF(s): Endothelium-derived relaxing factor(s)
EET(s): Epoxyeicosatrienoic acid(s)
eNOS/NOS III: Endothelial nitric oxide synthase
EOX: Cytochrome P_450_ epoxygenases
ER: Endoplasmic reticulum
ERK1/2: Extracellular signal-regulated kinases 1 and 2
ERK5: Extracellular-signal-regulated kinase 5
ET-1: Endothelin-1
ET_A_: Endothelin receptor A
ET_B_: Endothelin receptor B
GC: Guanylate cyclase
GCC: Glucocorticoid
GR: Glucocorticoid receptor
GTP: Guanosine-5′-triphosphate
HETE(s): Monohydroxyeicosatetraenoic acid(s)
H_2_O_2_: Hydrogen peroxide
H_2_S: Hydrogen sulfide
IK_Ca_: Intermediate conductance calcium-activated K^+^ channel
iNOS/NOS II: Inducible nitric oxide synthase
IP: Prostacyclin receptor
IP_3_: Inositol 1,4,5-trisphosphate
JNK: c-Jun N-terminal kinase
K_Ca_: Calcium-activated K^+^ channel
L-Arg: L-arginine
L-Cis: L-cysteine
L-NAME: N^G^-nitro-L-arginine methyl ester
LOX: Lipoxygenase
MAPK: Mitogen-activated protein kinase
ML: Membrane lipids
3MST: 3-Mercaptopyruvate sulfuresterase
mtNOS/NOS IV: Mitochondrial nitric oxide synthase
NADPH: Nicotinamide adenine dinucleotide phosphate
NF-*κ*B: Nuclear factor kappa B
nNOS/NOS I: Neuronal nitric oxide synthase
NO: Nitric oxide
NOS: Nitric oxide synthase
NOX: NADPH oxidase
NO_*x*_: Nitrate/nitrite
PG: Prostaglandin
PGI_2_: Prostacyclin
PIP_2_: Phosphatidylinositol 4,5-bisphosphate
PK: Protein kinase
PLC: Phospholipase C
p38MAPK: p38 mitogen-activated protein kinase
R: Receptor
ROS: Reactive oxygen species
SHR: Spontaneously hypertensive rats
SK_Ca_: Small conductance calcium-activated K^+^ channel
SOD: Superoxide dismutase
SR: Sarcoplasmic reticulum
TP: Thromboxane/prostaglandin endoperoxide receptor
TXA_2_: Thromboxane A_2_
VSM: Vascular smooth muscle cell
WKY: Wistar-Kyoto rats
XX: Other, not specifically mentioned substances which may produce hyperpolarization.
